# Author Correction: Enhancement of and interference among higher order multipole transitions in molecules near a plasmonic nanoantenna

**DOI:** 10.1038/s41467-022-29348-8

**Published:** 2022-03-24

**Authors:** Evgenia Rusak, Jakob Straubel, Piotr Gładysz, Mirko Göddel, Andrzej Kędziorski, Michael Kühn, Florian Weigend, Carsten Rockstuhl, Karolina Słowik

**Affiliations:** 1 Institute of Theoretical Solid State Physics, Karlsruhe Institute of Technology, 76131 Karlsruhe, Germany; 2 Institute of Physics, Faculty of Physics, Astronomy and Informatics, Nicolaus Copernicus University, Grudziadzka 5, 87-100 Torun, Poland; 3 Institute of Physical Chemistry, Karlsruhe Institute of Technology, 76131 Karlsruhe, Germany; 4 Institute of Nanotechnology, Karlsruhe Institute of Technology, 76021 Karlsruhe, Germany

**Keywords:** Nanophotonics and plasmonics, Atomic and molecular interactions with photons

Correction to: *Nature Communications* 10.1038/s41467-019-13748-4, published online 18 December 2019.

In the Acknowledgements section of this article the grant number relating to National Science Centre, Poland given for Karolina Słowik and Piotr Gładysz was incorrectly given as project no. 2016/23/G/ST3/0404 and should have been project no. 2016/23/G/ST3/04045. The original article has been corrected.

