# Supplementary material for: Enhancement of and interference among higher order multipole transitions in molecules near a plasmonic nanoantenna
Source: Nat Commun. 2019 Dec 18;10:5775. doi: 10.1038/s41467-019-13748-4 (PMC6920377; doi:10.1038/s41467-019-13748-4)
Supplement: Supplementary file 1 — Supplementary Information [file 41467_2019_13748_MOESM1_ESM.pdf]

Supplementary Information for:

Enhancement of and Interference among Higher Order Multipole Transitions in Molecules near Plasmonic Nanoantenna

Rusak *et. al.*

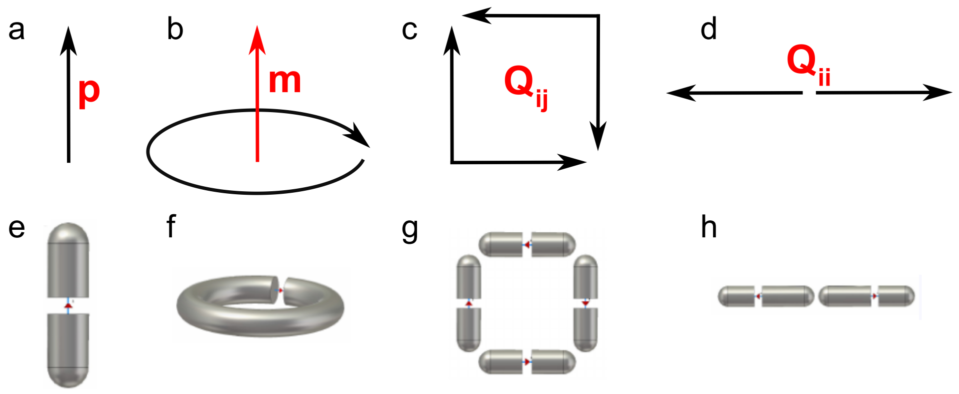

Supplementary Figure 1: Schematic representations and software implementations of different multipolar emitters. Top: The schematic representation of a. the electric dipole, b. magnetic dipole, c. off-diagonal and d. diagonal electric quadrupole emitters. Currents are displayed by black arrows. Bottom: e-h. implementation of the corresponding emitters in CST Microwave Studio.

Supplementary Note 1: Implementation of multipolar sources

Different molecular transition channels are modeled with different types of emitters implemented in the commercially available software package CST Microwave Studio (CST) within its frequency-domain solver [1]. The emitters are realized via oscillating electric currents in perfect electric conductors. The model of the electric dipole emitter is based on the principle of a Hertzian dipole [Supplementary Figure 1a,e]: A finite current oscillates over a tiny length causing an electric dipole moment parallel to the direction of the oscillation. The magnetic dipole emitter consists of a circular, oscillating electric current resulting in an oscillating magnetic moment parallel to the symmetry axis and perpendicular to the oscillating current [Supplementary Figure 1b,f]. To embody the electric quadrupole, it is necessary to represent the corresponding electric quadrupole tensor. This can be done in two steps. First, the off-diagonal elements can be represented by the canonical electric quadrupole depiction of four alternating charges placed in the corners of a square [Supplementary Figure 1c,g]. In CST, this approach is realized via four oscillating currents pointing to/away from the imagined charges. This structure corresponds to a quadrupole tensor with $Q_{ij} = 0$ if $i = j$ and $Q_{ij} = Q_{ji}$. We refer to this emitter as the "off-diagonal electric quadrupole emitter" (off- diag. EQ). Second, the diagonal elements can be accounted for by a linear arrangement of four charges (two of each kind), while the two charges in the center are of the same kind and closely spaced [Supplementary Figure 1d,h]. Two opposing, linearly aligned currents are used in CST to realize this case. This corresponds to a quadrupole tensor defined via the main diagonal with $Q_{ii} \neq 0$, and is later referred to as the "diagonal" electric quadrupole emitter (diag. EQ). The dimensions of the implemented emitters are on a length scale of a few Å and, therefore, are much smaller than the operational wavelength in the visible. This is an essential requirement, since the emitters must be operated far away from their intrinsic resonances.

To test the quality of the emitters, we performed a multipolar expansion of the electric field $\mathbf{E}(\mathbf{r})$ at the emission wavelength of the considered molecule at $\lambda = 553$ nm. For this, we use the scattering coefficients a_{nm} and b_{nm} as defined in Ref. Muhlig2011. The scattering coefficients are the multipole moments in spherical coordinates and can be transformed into Cartesian multipole moments for easier interpretation. The Cartesian electric and magnetic dipole moments ($n = 1$) are given by

$$\mathbf{p} = \begin{pmatrix} p_x \\ p_y \\ p_z \end{pmatrix} = C_0 \begin{pmatrix} a_{11} - a_{1-1} \\ i(a_{11} + a_{1-1}) \\ -\sqrt{2}a_{10} \end{pmatrix}, \mathbf{m} = \begin{pmatrix} m_x \\ m_y \\ m_z \end{pmatrix} = cC_0 \begin{pmatrix} b_{11} - b_{1-1} \\ i(b_{11} + b_{1-1}) \\ -\sqrt{2}b_{10} \end{pmatrix}, \quad (1)$$

with the constant $C_0 = i\sqrt{6\pi}\epsilon_0 k^{-1}$, where k is the wave number. The electric quadrupole moment (n =

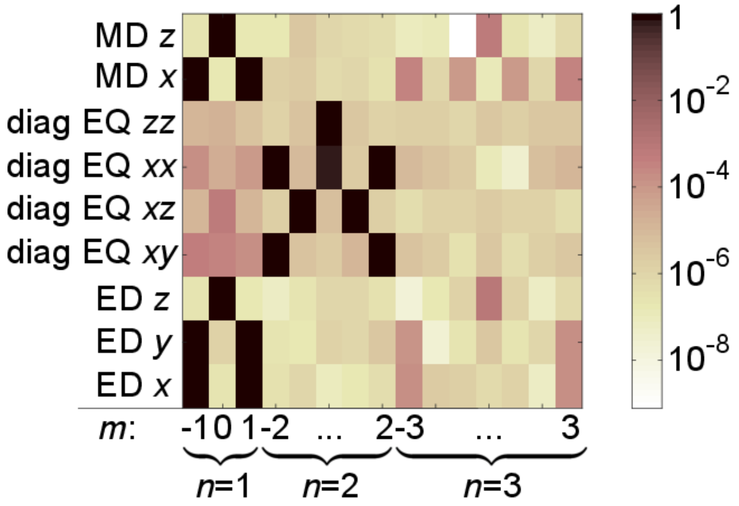

Supplementary Figure 2: Absolute square values of the multipolar coefficients. We only show $|a_{nm}|^2$ for the electric emitters and $|b_{nm}|^2$ for the magnetic dipole emitter in different orientations at a free-space wavelength of $\lambda = 553$ nm. The lower-case letters indicate the orientations of the emitters.

2) can be calculated from

$$\mathbf{Q} = \begin{pmatrix} Q_{xx} & Q_{xy} & Q_{xz} \\ Q_{yx} & Q_{yy} & Q_{yz} \\ Q_{zx} & Q_{zy} & Q_{zz} \end{pmatrix} \tag{2}$$
$$= D_0 \begin{pmatrix} i(a_{22}+a_{2-2}) - \frac{i\sqrt{6}}{2}a_{20} & a_{2-2}-a_{22} & i(a_{2-1}-a_{21}) \\ a_{2-2}-a_{22} & -i(a_{22}+a_{2-2}) - \frac{i\sqrt{6}}{2}a_{20} & a_{2-1}+a_{21} \\ i(a_{2-1}-a_{21}) & a_{2-1}+a_{21} & i\sqrt{6}a_{20} \end{pmatrix},$$

with $D_0 = -\frac{i\sqrt{30\pi}\epsilon_0}{k^2}$. In Supplementary Figure 2 we plot the absolute square values of the scattering coefficients $|a_{nm}|^2$ for the electric emitters and $|b_{nm}|^2$ for the magnetic dipole emitter. We do not show the contribution of the magnetic multipolar coefficients b_{nm} to the multipolar expansion of the electric emitters, since they are negligible, and analogously for the magnetic dipole emitter and the coefficients a_{nm}. We rescale the regarded quantities for the maximum value for all emitters in all orientations to be 1. An electric dipole emitter oriented in the x-direction has a dipole moment $p = p_x \sim a_{11} - a_{1-1}$. This is confirmed by our results: in Supplementary Figure 2 , the only significant contributions to this emitter (ED x) stem from $|a_{1-1}|^2$ and $|a_{11}|^2$. The other emitters in different orientations can be attributed to their corresponding Cartesian multipole moments, accordingly.

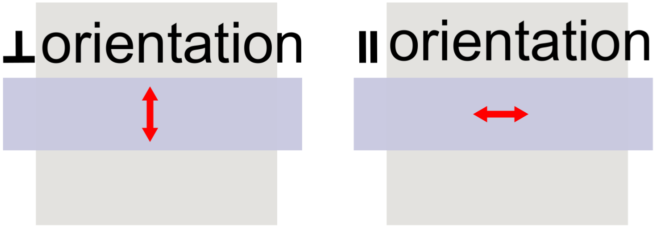

Supplementary Figure 3: Two characteristic orientations of the emitters relative to the patches of the nanoantenna. These basic orientations correspond to a source, indicated by the red double-arrow, perpendicular or parallel to the patches.

Supplementary Note 2: Field distributions

The transition rates Γ are studied in function of the source orientation. For this purpose the total, vectorial, complex electromagnetic fields around the nanoantenna must be evaluated at the source position, which is a numerically demanding task. To reduce the complexity we make use of the fact that an arbitrarily oriented source can be decomposed into a superposition of corresponding sources of basic orientations. For dipole moments and for sources representing the diagonal components of the quadrupole tensor (see above) the basic orientations are (Supplementary Figure 3)

1. parallel to the patches along the x, or equivalently along the y direction,
2. perpendicular to the patches along the z direction.

For sources that correspond to the off-diagonal elements of the quadrupole, the basic orientations are

1. parallel to the patches in the xy plane aligned with the x and y axes,
2. perpendicular to the patches equivalently in the xz or yz plane.

For each type of source of normalized strength and of each basic orientation we calculate the distribution of both the electric and the magnetic fields around the nanoantenna, using a frequency-domain solver as discussed in section *Implementation of multipolar sources* of this *Supporting Information*. Consequently, the complex values of the spatial field components of the resulting fields are extracted. Since the multipolar sources are designed to act as point sources, but molecular transitions can hardly be pinpointed on sub-molecular dimensions, we replaced the field inside the innermost cubic 1 nm^3 around the source by interpolated values. These interpolated values were generated by the Matlab internal function `interp3` based on the complete extracted fields in the cubic 1000 nm^3 surrounding the source. Due to the field interpolation, the generated field values are not distorted by the actual shape of the different multipolar sources as shown in Supplementary Figure 1 and potential numerical instabilities are also counteracted. The required field gradients are generated and processed in an analog fashion and finally the field values and gradients at the source position are calculated.

To model the composite response of such an intricate source representing a complicated actual molecular transition characterized by all its transition multipolar moments, we superpose the calculated complex electromagnetic field values and gradients with weights according to the ratio of the strengths of the corresponding transition multipolar moments. Due to the enhancement of the different transition channels through the nanoantenna and its fixed orientation, these superposition weights must be modified as the molecule is rotated with respect to the nanoantenna. This spatial-orientation-specific weight modification does not require completely new field-distribution simulations, since solely the superposition of the sources of basic orientations must be adjusted accordingly. The thusly calculated fields and gradients enter Eqn. 1 for different rotation angles of the molecule around the different spatial axes by means of Eqn. 2 leading ultimately to the plots displayed in Fig. 3 of the main text.

Supplementary Note 3: Purcell enhancement

Radiative and total Purcell enhancement factors calculated for different multipolar sources in basic orientations described above are shown respectively in Supplementary Figures 4 and 5.

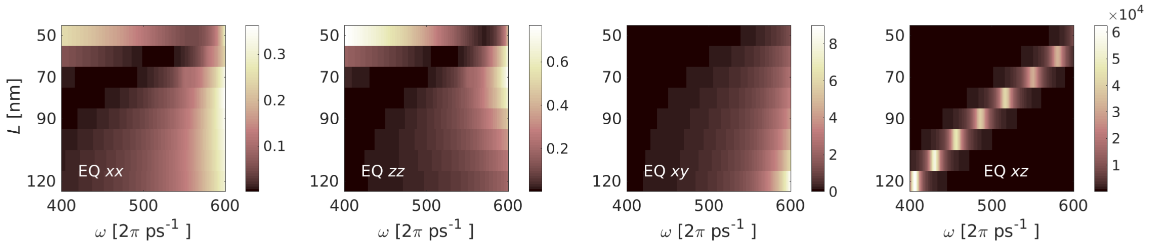

Supplementary Figure 4: Purcell enhancement of power radiated into far field by different multipolar sources in different basic orientations with respect to the nanoantenna discussed in this work.

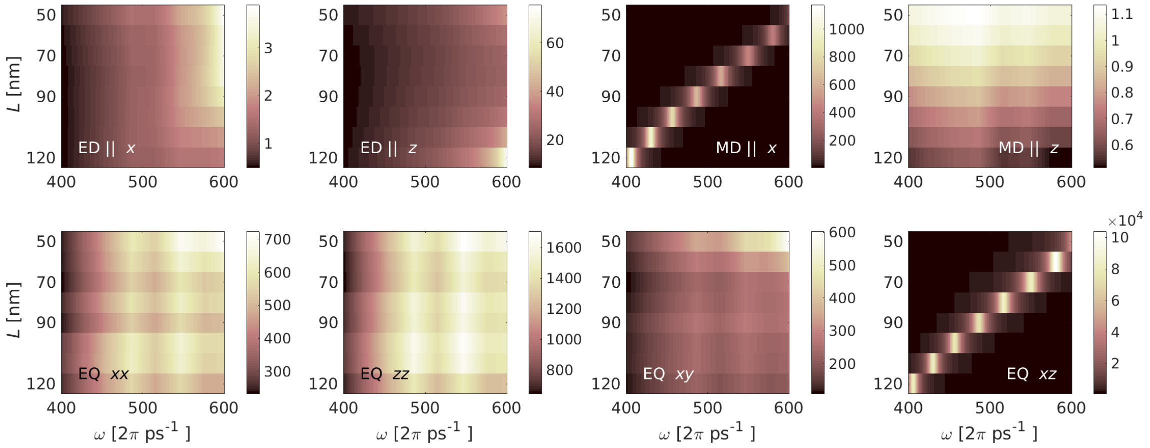

Supplementary Figure 5: Purcell enhancement of total power emitted by different multipolar sources in different basic orientations with respect to the nanoantenna discussed in this work.

Supplementary References

[1] CST MICORWAVE STUDIO 3D EM Simulation Software. https://www.cst.com/products/cstmws. Accessed: 2015-06-30.

[2] Mühlig, S., Menzel, C., Rockstuhl, C., and Lederer, F. Multipole analysis of meta-atoms, *Metamaterials* 5, 64–73 (2011).
